# HEALTH CARE UTILIZATION OF AGING INDIVIDUALS EXPERIENCING CHRONIC HOMELESSNESS: A CASE STUDY APPROACH

**DOI:** 10.1093/geroni/igae098.0398

**Published:** 2024-12-31

**Authors:** Russell Berg

**Affiliations:** Harborview Medical Center UW Medicine, Seattle, Washington, United States

## Abstract

The population of individuals experiencing homelessness in the United States is both growing and aging. Late baby boomers are overrepresented amongst those experiencing chronic homelessness and this cohort is now of geriatric age. Many challenges interfere with providing medical care to this group including mental health, substance use disorders, carceral history, lack of surrogate decision makers and dementia. In some cities, the COVID 19 pandemic led to transition of congregate emergency homeless shelters to hotels, providing an opportunity to co-localize enhanced services such as embedded medical services. This session will present three medical cases from a low barrier, harm reduction focused hotel shelter for chronically homeless individuals. The cases focus on selected individuals with high hospital utilization and demonstrate how the hotel shelter model with embedded medical clinic can function as a “medical home” reducing unnecessary service utilization while pursuing placement for those requiring a higher level of care. While residing at the shelter, important medical conditions such as dementia and Parkinson’s disease can be diagnosed that qualify residents for additional benefits. For some patients with severely limited life expectancy, the hotel shelter space can provide a setting for patient centered palliative care and even home hospice services. The challenges to providing care for an aging population experiencing homelessness require novel models for the provision of health care and hotel shelters with embedded medical care can fill the role of “medical home”.

